# Anoikis-related signature identifies tumor microenvironment landscape and predicts prognosis and drug sensitivity in colorectal cancer

**DOI:** 10.7150/jca.91627

**Published:** 2024-01-01

**Authors:** Yu-Biao Pan, Wang-jin Xu, Meng-sha Huang, Yan-di Lu, Yi-jing Zhou, Ya Teng, Jian-bin Gong, Xin-yu Fu, Xin-li Mao, Shao-wei Li

**Affiliations:** 1 Taizhou Hospital of Zhejiang Province, Zhejiang University, Linhai, China; 2 Taizhou Hospital of Zhejiang Province Affiliated to Wenzhou Medical University, Linhai, China; 3 Hospital of Huangyan affiliated to Wenzhou Medical University, Huangyan, Zhejiang, China; 4 Department of Gastroenterology, Taizhou Hospital of Zhejiang Province affiliated to Wenzhou Medical University, Linhai, Zhejiang Province, China; 5 Key Laboratory of Minimally Invasive Techniques & Rapid Rehabilitation of Digestive System Tumor of Zhejiang Province, Taizhou Hospital of Zhejiang Province affiliated to Wenzhou Medical University, Linhai, Zhejiang Province, China; 6 Institute of Digestive Disease, Taizhou Hospital of Zhejiang Province Affiliated to Wenzhou Medical University, Linhai, China

**Keywords:** Colorectal cancer, anoikis, signature, TME, prognostic model, drug predication

## Abstract

**Background:** Anoikis, a mechanism of programmed apoptosis, plays an important role in growth and metastasis of tumors. However, there are still few available comprehensive reports on the impact of anoikis on colorectal cancer.

**Method:** A clustering analysis was done on 133 anoikis-related genes in GSE39582, and we compared clinical features between clusters, the tumor microenvironment was analyzed with algorithms such as “Cibersort” and “ssGSEA”. We investigated risk scores of clinical feature groups and anoikis-associated gene mutations after creating a predictive model. We incorporated clinical traits to build a nomogram. Additionally, the quantitative real-time PCR was employed to investigate the mRNA expression of selected anoikis-associated genes.

**Result:** We identified two anoikis-related clusters with distinct prognoses, clinical characteristics, and biological functions. One of the clusters was associated with anoikis resistance, which activated multiple pathways encouraging tumor metastasis. In our prognostic model, oxaliplatin may be a sensitive drug for low-risk patients. The nomogram showed good ability to predict survival time. And SIRT3, PIK3CA, ITGA3, DAPK1, and CASP3 increased in CRC group through the PCR assay.

**Conclusion:** Our study identified two distinct modes of anoikis in colorectal cancer, with active metastasis-promoting pathways inducing an anti-anoikis subtype, which has a stronger propensity for metastasis and a worse prognosis than an anoikis-activated subtype. Massive immune cell infiltration may be an indicator of anoikis resistance. Anoikis' role in the colorectal cancer remains to be investigated.

## Introduction

Colorectal cancer (CRC) accounts for 10% of all malignancies and cancer-related deaths globally^1^. According to data, the number of newly diagnosed CRC cases exceeded 1.9 million in 2020 2, 3. 20% of CRC patients develop metastatic disease, and 40% return following local treatment. Metastatic CRC has a less-than-20% 5-year survival rate 4. However, with our improving insights into the diversity and complexity of the tumor microenvironment (TME), immunotherapy has achieved substantial and long-lasting results in the treatment of CRC^5^. Although research on CRC continues to advance, the incidence of CRC is still reported to be on the rise^6^. Previous studies have also predicted that deaths from CRC will increase substantially by 2035^7^. Therefore, the exploration of new biomarkers has important clinical implications for clarifying the prognosis and most appropriate treatment of CRC.

Anoikis is a specific programmed apoptosis initiated by the detachment of cells from the extracellular matrix (ECM), which is critical in organism development, disease development and tumor metastasis^8^. Several previous studies have elucidated the various mechanisms employed by cancer cells to eliminate anoikis and promote metastasis and invasion, including reactive oxygen species (ROS) 9, non-coding RNAs^10^ and signaling pathways^11^.

The resistance of aggressive tumor cells to anoikis represents a pivotal aspect of tumor progression and a potential target for therapeutic intervention. For example, Ye et al. found that CTNNB1 transcription, which was induced by nuclear MYH9, facilitated anoikis resistance and metastasis in gastric cancer cells^12^. Furthermore, the HBXIP/Nrf2 feedback loop promotes breast cancer anoikis resistance by maintaining redox homeostasis and inhibiting JNK1 activation^13^. Furthermore, Syndecan-2 overexpression in melanoma cells activates PI3K/Akt and ERK signaling, underscoring the ECM's role in anchorage independence and cancer cell invasion^14^. Additionally, in cholangiocarcinoma, metformin-induced AMPK activation sensitizes cells to anoikis, warranting further investigation into AMPK's role in anoikis resistance and metastasis^15^.

There has also been increasing interest in CRC, where several genes involved in fatty acid oxidation, particularly the rate-limiting enzyme CPT1A, are upregulated in suspension-grown CRC cells. CPT1A in CRC cells is able to mediate the scavenging of ROS clusters, a mechanism that is important for imbuing cellular resistance to anoikis^16^. It is suggested that CPT1A is a new potential target for the treatment of metastatic CRC. Integrins exert their influence on cellular resistance to anoikis through interactions with various molecules, particularly EGFR and oncogenes, as well as synergistic actions with growth factors^17^. By regulating multiple signaling pathways, including ERK/Akt, MAPK, and AKT, integrins facilitate cell survival in an environment detached from the extracellular matrix^18^. This occurs through the coordination of integrin interactions with diverse molecular partners, highlighting their pivotal role in maintaining cell viability when detached from the substrate^19^. And in recent years, it has also been found that increased co-binding of EGFR with integrin-α2β1/-α5β1 on the surface of anoikis-resistant cells regulates anoikis resistance in CRC cells^20^. Furthermore, ATP enzyme inhibitor 1 (IF1), a physiological inhibitor of ATP synthase, is overexpressed in a significant proportion of malignancies that contribute to metabolic alternations and tumor development, and the overexpression of mitochondrial IF1 prevents metastatic disease in CRC through promoting anoikis and neoplasm infiltration in NK cells^21^. A study revealed that KLF5 is a novel prognostic biomarker for CRC and may play a role in maintaining anoikis resistance in CRC cells^22^.

Several anoikis-related genes have been linked to CRC metastasis and prognosis in previous reports, but the anoikis signature has not been comprehensively studied. This study evaluated CRC transcriptome data to clarify the role of anoikis in CRC, indicating that its mechanism of action may influence prognosis. Our findings may help us understand CRC and its therapy by complementing the role of anoikis.

## Methods

### Download and processing of Data

The study data were partly obtained in public GEO databases. We first retrieved the search term "colorectal cancer" in the GEO databases. Finally, we download GSE39582, GSE72970, and GSE38832 by the R package “GEOmirror” ('http://raw.githubusercontent.com/jmzeng1314/GEOmirror/master'). We opted to analyze each dataset separately to avoid biological differences from merging.

The data cleaning process for the microarray data was as follows: 1) samples with missing survival time or survival status were excluded; 2) survival time was restricted to <30 days; 3) if the dataset was not log2 processed, log2+1 normalization was performed to avoid negative expression values; 4) intra-group correction was conducted using the removeBatchEffect function of the limma package^23^. The final GSE39582 dataset had 575 cases: 556 tumors and 19 normals. GSE38832 included 119 samples and GSE72970 124.

We used “tcgabiolinks (version) 2.24.3”^24^ to download the TCGA-COAD STAR-Counts and extract TPM values for subsequent analyses. For the TCGA-COAD dataset, we excluded patients with missing data on the survival time or with a survival time <30 days. Ultimately, 424 patients were included. The “cBioPortalData” 25 package was used to download the TCGA-COAD MSI information.

Considering the advantage of the GSE39582 sample size in the included dataset and the rich clinical information (including gender, age, TNM stage, CpG Island Methylator Phenotype (CIMP), chromosome instability (CIN),and mismatch repair (MMR)), we used it as the primary analysis dataset, while the TCGA was used to supplement the analysis of the MSI status as well as anoikis gene mutations, and the other two datasets from GEO were mainly used for external validation of the model.

### Identification of anoikis-related clusters

We collected 133 anoikis-related genes from Harmonizome (https://maayanlab.cloud/Harmonizome/) for anoikis (Supplementary Table 1) and extracted the expression of 133 genes in 556 CRC patients in the GSE39582 dataset using “ConsensusClusterPlus”[26]with the following parameters: clusterAlg= “pam”, distance=“spearman”, repeated 1000 times to ensure the reliability of clustering. We used a principal component analysis (PCA) for a dimensionality reduction analysis. We also analyzed the differences in survival time between the clusters and the correlation with chemotherapy by a Kaplan-Meier analysis. In addition, we compared TNM stage, tumor location, CIMP, CIN, and MMR between the groups, and the chi-square test was used to determine whether there were any significant differences between the clusters. The outcomes were presented using stacked bar graphs.

### Differential genes and functional enrichment analyses

We subsequently identified differentially expressed genes between clusters A and B using the limma package, selecting |logFC|> 1 and adjutsted p-value <0.05 as the threshold. Genes that met the threshold condition were considered as anoikis subtype differential genes. After obtaining the differential gene set, we performed Gene Ontology (GO) and Kyoto Encyclopedia of Genes and Genomes (KEGG) enrichment analyses using “clusterProfiler”, and the p-valueCutoff & qvalueCutoff were both set at 0.05.

Given that the contribution of non-differentially expressed genes to biological pathways cannot be denied, we performed a single-sample geneset enrichment analysis (ssGSEA)^27^ on 556 patients, and the dataset was selected as KEGG (c2.cp.kegg.v7.5.1.symbols.gmt) and hallmark (h.all.v7.5.1.symbols.gmt) (msigdb, gsea-msigdb.org/gsea/msigdb/). For the scoring results, the data were normalized by the Min-Max method, and the differential expression pathways were calculated by the limma package.The inter-cluster differences were displayed via heat map with an adjusted p-value 0.05.

### Immune infiltration analyses

To explore the differences in immune microenvironment between subtypes, we first assessed the immune, stromal and tumor purity via the “ESTIMATE”^28^. To specifically characterize immune cell infiltration, we used “CIBERSORT”^29^ to assess the differences in infiltration of 22 immune cells, with perm set to 1000. The ssGSEA was further applied to assess the abundance of “MDSC”, “macrophages” and “regulatory T cells”.

### Constructing and validating a prognostic model and evaluating prognostic performance

We took GSE39582 as the training dataset. Then univariate cox regression was first performed on 133 genes. Genes with statistical significance were included. Subsequently, we employed Lasso-penalized Cox regression and multifactorial Cox regression method to filter for prognostic genes and compute regression coefficients. Then we computed risk scores according to the expression of each gene using the following formula:

Risk score = [(Exp gene1 × coefficient gene1) + (Exp gene2 × coefficient gene2) +--+ (Exp geneN × coefficient geneN)]

We applied the “survminer” package to classify patients into high- and low-risk groups. Kaplan-Meier curves were used to compare survival between groups. Then time-dependent receiver operating characteristic curve(time-ROC) was utilized in the assessment of the predictive performance. Moreover, we conducted external validation of the risk score model on the data sates, GSE72970, GSE38832 and TCGA. To better portray the prognostic model, we analyzed the risk score in different clinical feature groups.

We used the “tools” package to depict the difference in the frequency of anoikis-associated mutations in the different risk groups, and the 2×2 chi-square test was used to calculate statistical significance. The "tmb" function of the "maftools"^30^ package was used to assess the tumor mutation burden (TMB), with box plots used to present the variation of TMB between risk groups. The "oncoPredict"^31^ package, a tool for drug sensitivity prediction, was used to evaluate the sensitivity of 196 drugs across patient risk groups. It employs the 'calcPhenoty' function with training from two datasets, CTRP-V2 and GDSC-V2, to predict drug sensitivity based on patient data. This approach ensures a robust model capturing genomic features and drug responses for comprehensive analysis.

We combined the prognostic model with clinical traits after Lasso regression analysis. We then screened the prognostic factors according to the “lambda.min” value by stepwise regression and judged the prognostic factors according to the minimum Akaike information criterion (AIC) value. The final results from the aforementioned research were utilized to generate nomogram predicting the OS of CRC patients. The model's stability was tested using time-ROC and the calibration curve.

### Quantitative Real-Time PCR

In this study, we collected 78 colon samples (39 cancer tissue samples and 39 corresponding normal tissue samples) obtained from surgical procedures or endoscopic examinations conducted at Taizhou Hospital in Zhejiang Province, China, between 2016 and 2021. Cellular RNA was extracted using Trizol reagent (Thermo Fisher Scientific). Subsequently, following the manufacturer's recommendations, reverse transcription into cDNA was carried out using the Prime ScriptTM RT kit (Takara Biotechnology Co., Dalian, China). The cycle threshold (CT) values for each group were determined, and the expression levels of six anoikis-related genes (SIRT3, PIK3CA, ITGA3, DAPK1,PAK1 and CASP3,) were calculated using the 2^-ΔΔCT^ method with three independent replicates. Primer sequences are available in the Supplementary Table 2. All samples obtained from real-world sources were acquired with the approval of the the institutional review committee of Taizhou Hospital of Zhejiang Province Affiliated to Wenzhou Medical University, and strict adherence to relevant regulations and ethical guidelines was observed.

### Statistical analysis

Statistical analyses and data visualization were performed using GraphPad Prism version 5.0 (La Jolla, CA, USA) and R 4.1.2 (R Core Team, Massachusetts, USA). Prognostic differences among sample groups were assessed using the logrank test. To evaluate the significance of results, we conducted a Kruskal-Wallis test for three groups and employed the Wilcoxon test for pairwise comparisons between two groups. In all analyses, p-values less than 0.05 were considered statistically significant.

## Results

### The workflow of our study

**Figure 1:**
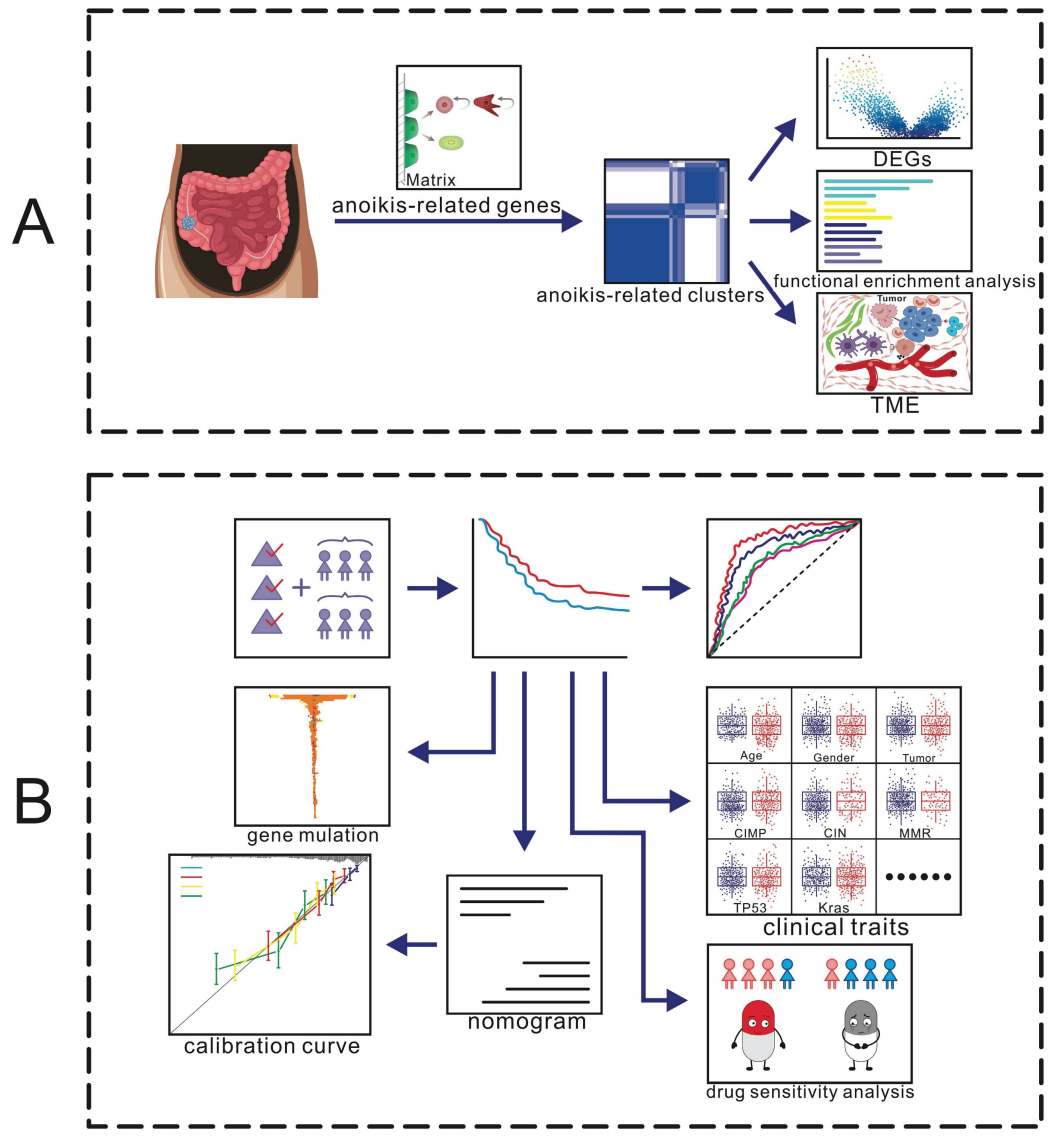
** Diagrammatic representation of the flow of the study.** (A) Identification of anoikis-related clusters and a tumor microenvironment analysis. (B) Construction and validation of a gene prognostic model.

We collected GSE39582 data and performed a cluster analysis based on 133 anoikis-related genes to obtain 2 clusters, based on which we performed differential gene, functional enrichment and TME analyses (Fig. 1A). To identify prognosis-related genes, we used Cox regression and Lasso-penalized Cox methods, ultimately identifying six genes. According to the expression of these six genes, patients were categorized as high- or low-risk. Then we explored the anoikis-related gene mutations in the subgroups and assessed the risk score of several clinical traits. A clinical prognostic model was developed, a prognostic nomogram was produced and validated, and the calibration curve was examined. Furthermore, we collected colon cancer samples and utilized RT-PCR to validate the expression of the 6 model genes (Fig. 1B).

### Identification of anoikis-related clusters

**Figure 2:**
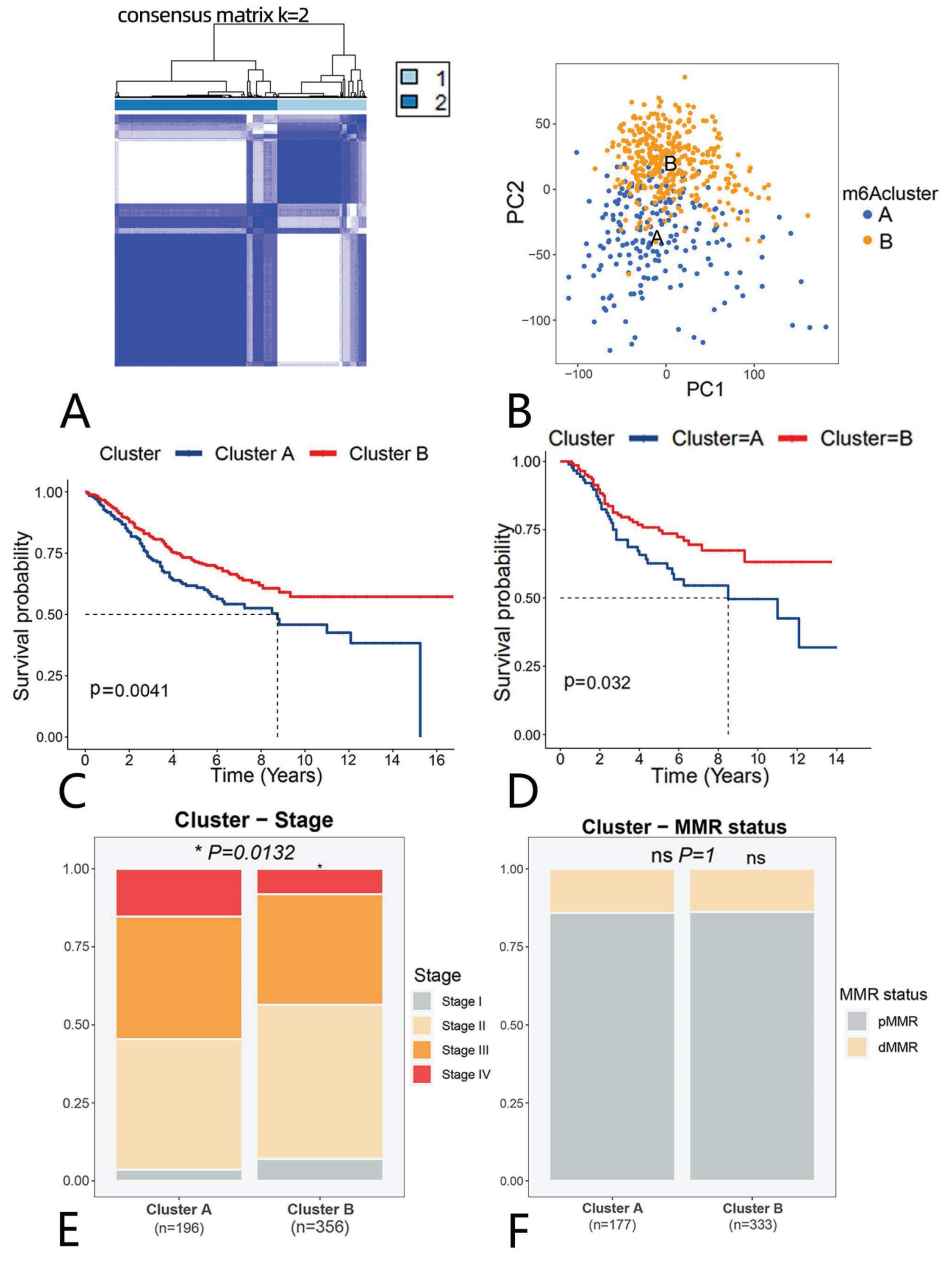
** Identification of anoikis-related clusters and the analysis of their clinical relevance.** (A) A cluster analysis of the GSE39582 dataset. (B) A PCA of anoikis-related clusters. (C) Survival curves. Blue (cluster A); red (cluster B). (D) Survival curves in chemotherapy-treated patients. Blue(cluster A); red(cluster B) (E) Proportion of each stage in clusters A and B. Grey represents stage I, yellow stage II, orange stage III and red stage IV.(F)Proportion of MMR status in clusters A and B.

We clustered GSE39582 using "ConsensusClusterPlus", according to 133 anoikis-related genes, obtaining 2 clusters (clusters A and B; k=2); cluster A contains 196 patients, while cluster B contains 360 patients. (Fig. 2A). We then used a PCA to downscale the both clusters, thus clearly distinguishing the clusters (Fig. 2B). As presented in Fig. 2C the survival analysis showed cluster B outperformed cluster A, suggesting that different anoikis subtypes may have different modes of action in CRC. Survival of CRC patients is closely related to treatment and tumor stage. We then evaluated whether or not chemotherapy affects the survival in different subgroups. We performed separate survival analyses for chemotherapy-treated and non-chemotherapy-treated patients in clusters A and B. As shown in Fig. 2D, the survival of chemotherapy-treated patients in cluster B was significantly better than that of chemotherapy-treated patients in cluster A. However, in the subgroup analysis without chemotherapy, we did not see a significant difference (Supplementary Fig. 2A). We then performed a separate chemotherapy-treated and non-chemotherapy-treated subgroup analysis within the two clusters, with no survival differences noted within either cluster (Supplementary Fig. 2B, C). These findings suggest that may be some difference between clusters A and B that allow patients to benefit from chemotherapy.

We then compared the two clusters' clinical stages. Stages I and II accounted for a larger proportion of cluster B than cluster A, while stages III and IV accounted for a smaller proportion (Fig. 2E). Furthermore, other clinically relevant indicators, such as the MMR state, tumor location, CIMP status and CIN status, were not statistically different (Fig. 2F, Supplementary Fig. 2D-F).

### Identification of differential genes and a functional enrichment analysis in anoikis-related clusters

**Figure 3:**
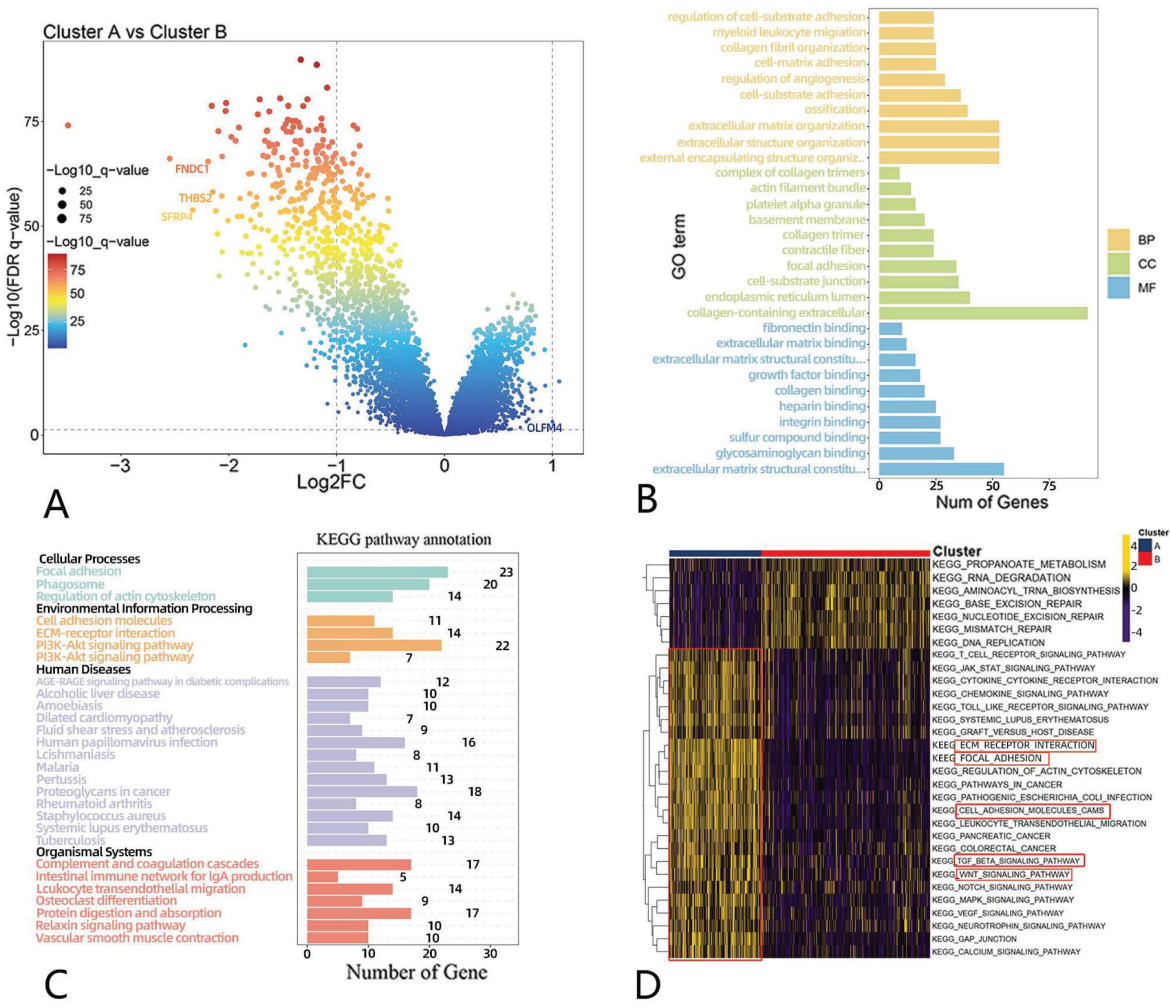
** Differential gene and functional enrichment analyses of anoikis-related clusters.** (A) Volcano map showing differential genes between clusters A and B. (B) A GO analysis of differential genes. (C) A KEGG analysis of differential genes. (D) Differential pathways. Yellow represents the upregulated pathways, and black represents the downregulated pathways.

The anoikis-related clusters showed significant clinically specific differences, suggesting that there may be differences in biological pathways between the two clusters. The volcano chart in Fig. 3A shows that 2 genes were upregulated, and 380 were downregulated. We noticed that FNDC1, THBS2 and SFRP4 had a low expression in cluster B, while the OLMF4 expression was elevated, all of which were involved in tumorigenesis and progression in previous reports (Fig. 3A). This may indicate the dysregulation of oncogene expression between clusters.

To further investigate the possible biological function of anoikis, we did a functional enrichment analysis. In the GO analysis, we found that “regulation of cell substrate adhesion”, “cell matrix adhesion”, “cell-containing extracellular matrix”, and “extracellular matrix binding” were enriched (Fig. 3B). A KEGG analysis showed that differential genes were mapped to “focal adhesion”, “ECM-receptor interaction”, and “PI3K-AKT signaling”, which are associated with tumor metastasis (Fig. 3C).

And we used ssGSEA approach to evaluate the differences in pathways between two clusters. Our findings showed that, in cluster A, “ECM-Receptor interaction”, “focal adhesion”, “NOTCH signaling pathway”, “WNT signaling pathway”, “MAPK signaling pathway”, and “TGF-β signaling pathway” were upregulated, while DDR-related pathways in cluster B were enriched, including “base excision repair”, “MISMATCH repair”, and “DNA replication” (Fig. 3D).

Additionally, we compared the hallmarks between the two clusters. The expression of hallmarks, such as NOTCH signaling, TGF-β signaling, WNT β-catenin signaling and angiogenesis, was up-regulated in cluster A (Supplementary Fig. 3A).

### The TME analysis

**Figure 4:**
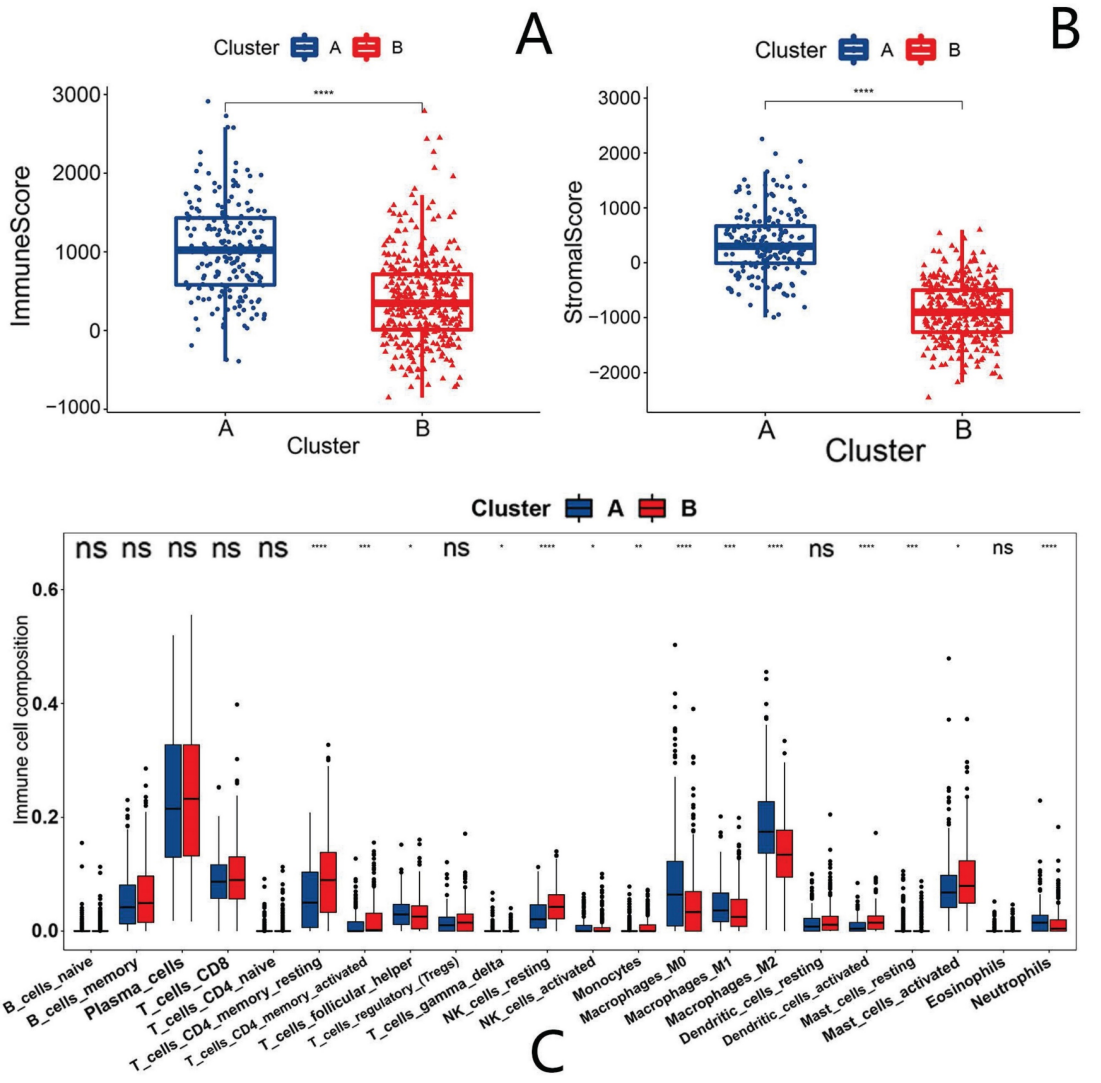
** Differences in the tumor microenvironment of anoikis-related clusters.** Blue represents cluster A, and red represents cluster B. (A) Immune score in anoikis-related clusters. (B) Stromal score in anoikis-related clusters. (C) The infiltration of 22 immune cell types Ns means “not statistically significant”; *p < 0.05; **p < 0.01; ***p < 0.001; ****p < 0.0001 (all significance designations that appear in this paper are minor criteria).

The TME has received increasing academic attention in CRC in recent years. Therefore, we also analyzed the differences in the TME between clusters A and B. We first evaluated the stromal and immune scores using “ESTIMATE” and found that the scores of cluster A were higher than those of cluster B (Fig. 4A, B). However, as for tumor purity, cluster A scored less than cluster B (Supplementary Fig. 3B). The results indicate immunological heterogeneity between clusters. We further assessed the immune cell differences via the CIBERSORT method and found that M2 macrophages, myeloid-derived suppressor cells (MDSCs) and regulatory T cells were higher in cluster A than cluster B (Fig. 4C). We then assessed the infiltration of macrophages and regulatory T cells using ssGSEA, and indeed, we found the elevated expression of these immune cells in cluster A compared with cluster B (Supplementary Fig. 3E).

### Construction of a risk model

**Figure 5:**
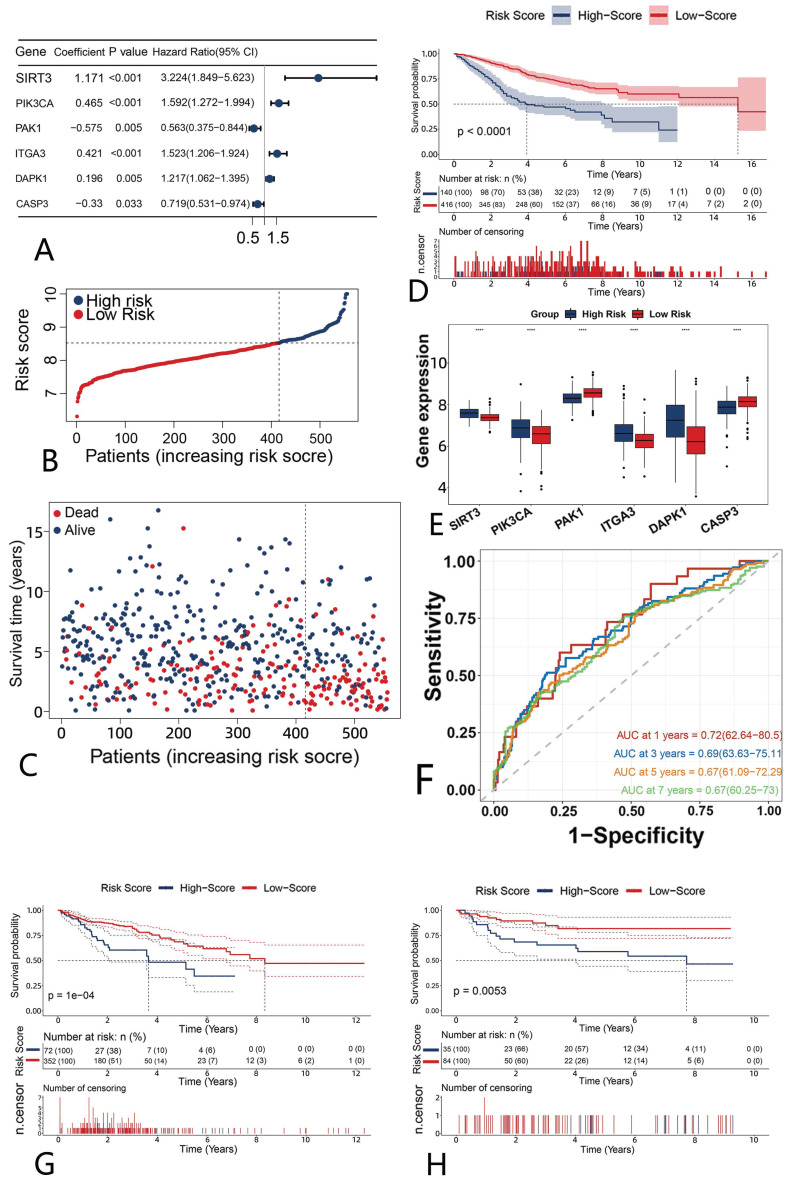
** The construction and evaluation of a prognostic model of six anoikis-related genes.** (A) Forest plot of the six genes (B) Risk scores of patients in GSE39582. Blue (high-risk); red (low-risk) (C) The survival status in the GSE39582. Red represents “dead”, and blue “alive”. (D) Survival differences in the high-risk and low-risk patients of GSE39582. (E)Expression of the six anoikis-related genes in high- and low-risk groups. Blue (high-risk); red (low-risk). (F) A time-ROC curve analysis of the risk model in GSE39582. (G-H) The Kaplan-Meier survival analysis of the external validation datasets. Blue ( high-risk); red (low-risk) G: TCGA-COAD; H: GSE38832

The above findings suggest that anoikis has a different mechanism in CRC. We then used univariate Cox regression to analyze anoikis-related genes and obtained 31 genes associated with the survival (Supplementary Fig. 3C). We performed a Lasso-penalized Cox regression analysis to further screen the six genes with a hazard ratio (HR) >1 (SIRT3, PIK3CA, ITGA3 and DAPK1, which we considered potential risk genes) and an HR <1 (PAK1 and CASP3, which we considered potential protective genes) (Fig. 5A). The risk score was then calculated with the formula:

Risk score= [(Exp SIRT3 × (1.171) + (Exp PIK3CA × (0.465) + Exp ITGA3 × (0.421) + Exp DAPK1 × (0.196) + Exp PAK1 × (-0.575) + Exp CASP3 × (-0. 33)]

Based on the best cut-off values, patients in the GSE39582 were classified into high- and low-risk categories, and we saw that the cut-off values differentiated surviving and dying patients (Fig. 5B, C). The Kaplan-Meier curves demonstrated that the low-risk group had a superior OS compared to the high-risk group (Fig. 5D). We next evaluated the expression of six genes in the two groups. It could be noticed that potential risk genes expressed more strongly in the high-risk group, while potential protective genes expressed more strongly in the low-risk group, further confirming the reliability of the screened genes (Fig. 5E).

Time-ROC curves indicated that the gene prognostic model demonstrated strong predictive ability, with areas under the curve (AUCs) of 0.72, 0.69, 0.67 and 0.67 predicting the OS at 1, 3, 5 and 7 years, respectively (Fig. 5F). We assessed the risk score as above in three external validation datasets (GSE38832, GSE72970 and TCGA). The results showed that the high-risk group had a much poorer prognosis than the low-risk group (Fig. 5G, H, Supplementary Fig. 3D), suggesting the stability of our constructed model.

### The correlation between risk score and clinical traits

**Figure 6:**
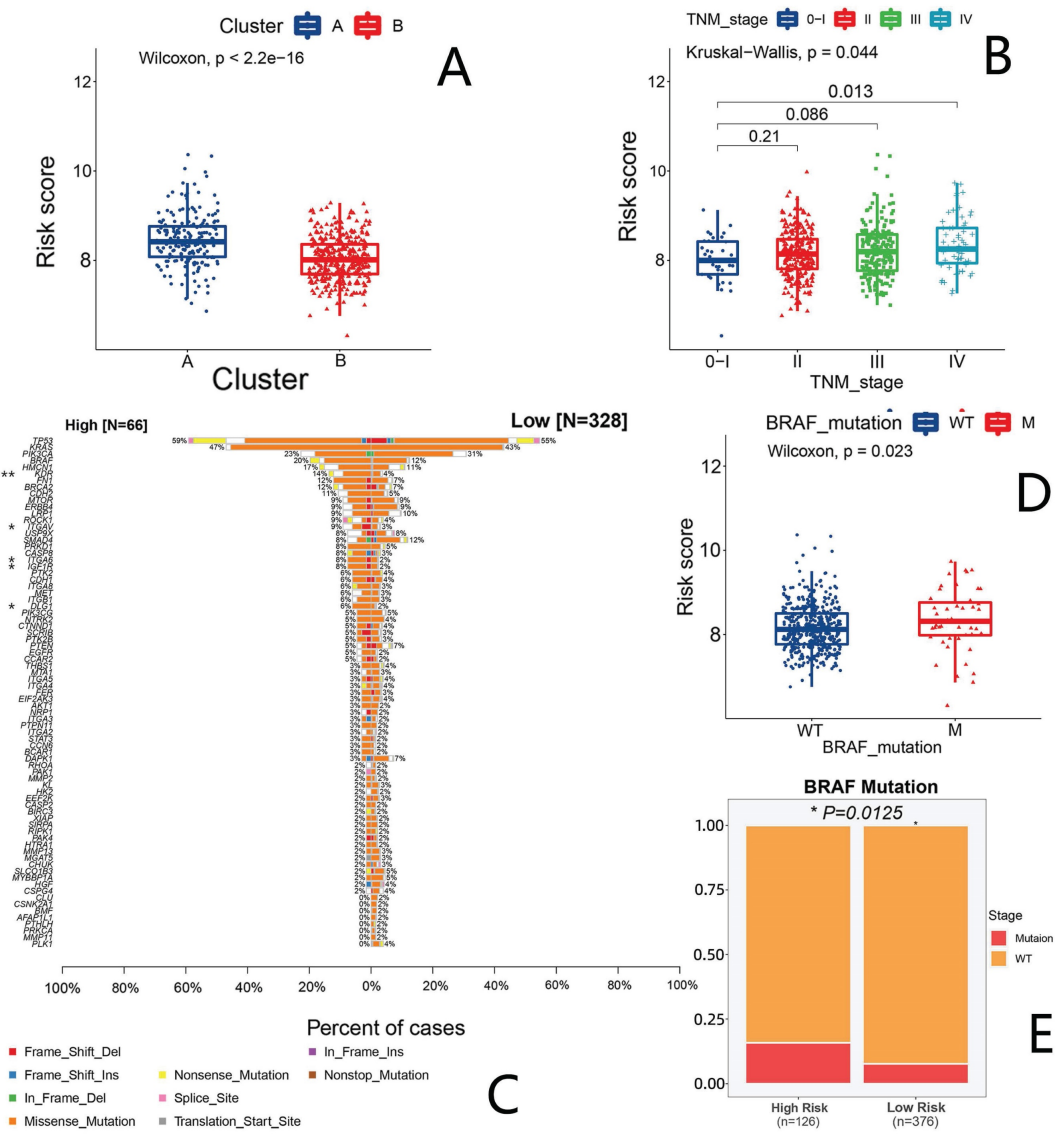
** The analysis of the clinical characteristics and gene mutations in the prognostic model.** (A) Differences in risk scores between clusters A and B. Blue (cluster A); red (cluster B). (B) Risk scores of patients in each stage. Blue represents stage 0/I, red stage II, green stage III and purple stage IV. (C) A waterfall diagram showing mutations in anoikis-related genes in the two groups. (D) Risk scores between BRAF-wild and BRAF-mutant groups. (E) Proportions of BRAF-wild and BRAF-mutation patients in the high- and low-risk groups.

After comparing the risk score scoring between patients in the clusters A and B, we found a higher risk score in cluster A with a poor prognosis, which is consistent with our finding that cluster A has a worse survival than cluster B and further indicating the reliability of our score (Fig. 6A).

We also contrasted the risk scores between patients with different stages. We found that patients in stage IV had the highest risk score, while those in stage I had the lowest (Fig. 6B). No statistically significant differences were observed in the comparison of risk scores among clinical characteristics, such as gender, age, CIMP status, CIN status, MMR status and tumor location (Supplementary Fig. 4A-F). Gene mutations attracts wide attention in the diagnosis and treatment of CRC. We therefore also evaluated the mutation status of these 133 anoikis-associated genes in the high- and low-risk groups. The results are shown in Fig. 6C, where we focused on the increased and statistically significant mutations among the KDR, ITGAV, IGF1R and ITGA6 genes, suspecting that the differences in the high- and low-risk groups might have been affected by gene mutation. Furthermore, we evaluated the risk of BRAF, TP53 and KRAS mutations separately and found that the risk score in BRAF mutation patients was higher than that in non-mutation patients (Fig. 6D), while there was no statistically significant difference in the scores among patients with and without TP53 and KRAS mutations (Supplementary Fig. 4G, H).

We further analyzed the proportion of BRAF mutation in the high- and low-risk groups, and found more patients with a BRAF mutation in the high-risk group than in the low-risk group (Fig. 6E). We therefore thought that BRAF mutations might be associated with a poor prognosis. We next verified in the TCGA that there was no statistically difference; however, patients in the BRAF mutation group showed a trend toward a higher risk than those in the non-mutation group (Supplementary Fig. 4I).

**Figure 7:**
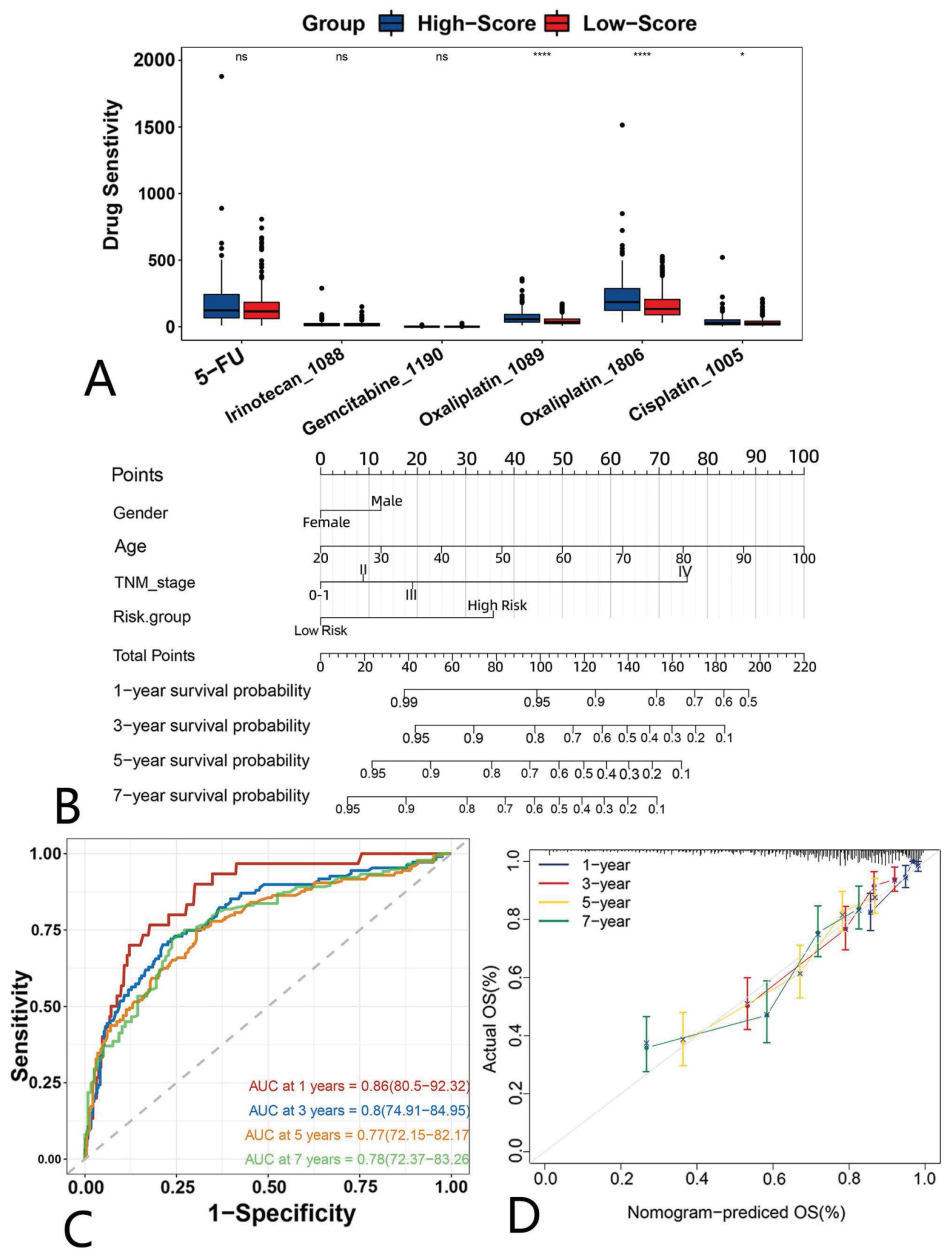
** A drug sensitivity analysis and nomogram construction.** Drug sensitivity differences between the high- and low-risk groups. Blue (high-risk); red (low-risk). (B) Nomogram for predicting the survival of CRC patients. (C) A time-dependent ROC of the nomogram. (D) Calibration curve of the nomogram.

In addition, we also assessed the drug sensitivity profile of the high- and low-risk groups. We found that most drugs' IC_50_ values were increased in the high-risk group, suggesting that there might be more chemotherapy-insensitive patients in the high-risk group (Supplementary Fig. 5A). We then focused specifically on several classes of drugs that are commonly applied in CRC and found that low-risk patients demonstrated superior drug sensitivity to oxaliplatin than high-risk patients, suggesting they may be a beneficial population for oxaliplatin. (Fig. 7A).

### Construction and validation of a predictive nomogram

We included clinical traits that are commonly considered prognostically relevant in clinical practice and are easily obtainable. The nomogram construction involved integrating the anoikis model risk group, as well as demographic factors such as age, gender, and TNM stage, determined through the lasso-combined stepwise approach (Fig. 7B). The Time-ROC analysis indicated AUCs of 0.86, 0.8, 0.77, and 0.78 for predicting OS at 1, 3, 5, and 7 years, respectively (Fig. 7C). Additionally, calibration curves demonstrated a general agreement between predicted and observed OS (Fig. 7D).

### Validation of the expression of 6 model genes in the real world

**Figure 8:**
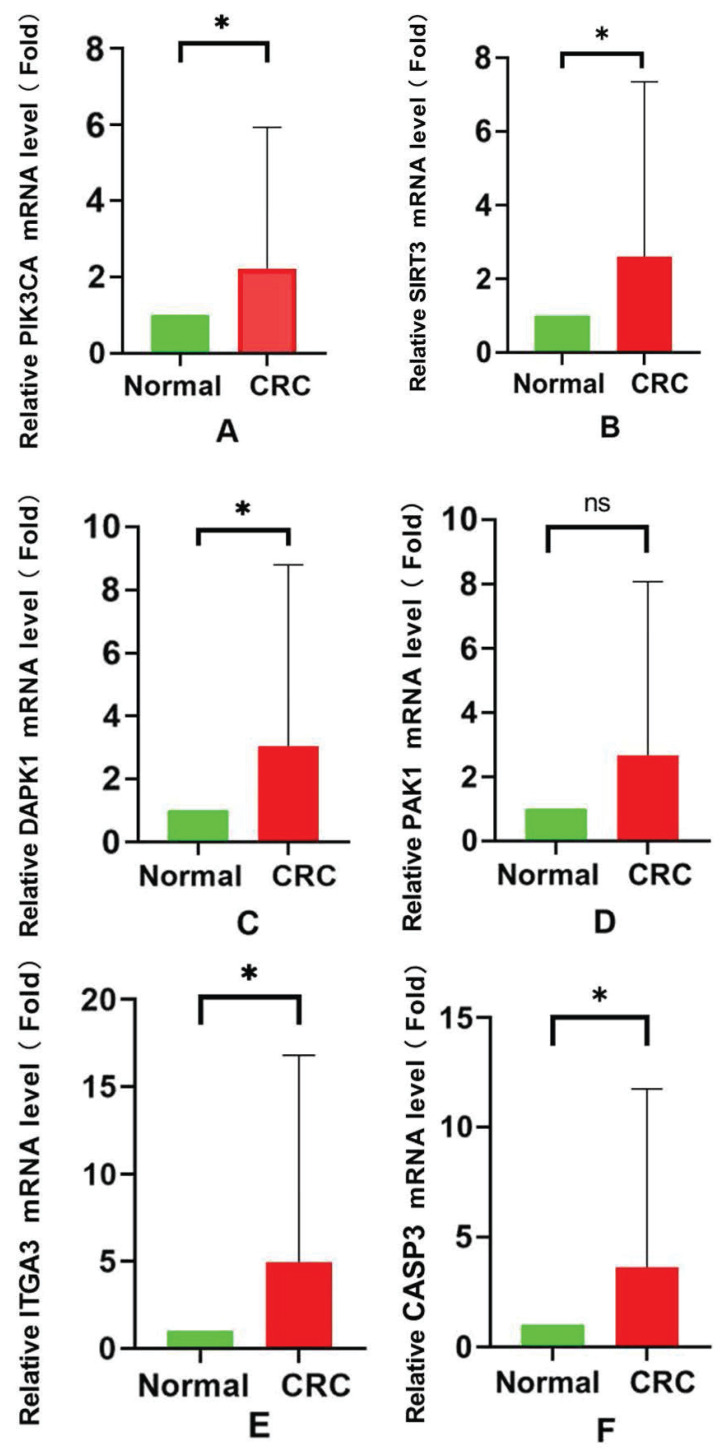
** The mRNA expression of 6 anoikis-related genes.** (A) PIK3CA; (B) SIRT3; (C) DAPK1; (D) PAK1; (E) ITGA3; (F) CASP3.

To confirm the successful induction of the 6 model genes (SIRT3, PIK3CA, ITGA3, DAPK1, PAK1, and CASP3), we gathered a set of 39 colon cancer tissue samples. The RT-PCR assay results clearly demonstrated a significant increase in mRNA expression, for SIRT3, PIK3CA, ITGA3, DAPK1, and CASP3, within the CRC group (Fig. 8A-F).This heightened expression indicted the reliability of the gene selection.

## Discussion

Anoikis is a specific type of cell death that has received much attention in recent years^32^. Several studies have elucidated the various mechanisms employed by cancer cells to eliminate anoikis and promote their metastasis and invasion. Therapeutic approaches targeting anoikis have been initiated in some cancers^33^. In studies on CRC, genes such as CPT1A, IF1 and KLF5 have been found to be involved in anoikis resistance^16^, ^21^, ^22^. However, an anoikis signature in CRC has not been systematically identified. Therefore, our study explored the role of anoikis in the development and metastasis of CRC through bioinformatics.

In the study, the prognosis of cluster A was worse than that of cluster B, including in patients who also received chemotherapy. This suggests that there is heterogeneity in the role of anoikis in CRC patients, which may benefit patients receiving chemotherapy. Anoikis resistance is an important feature of tumor metastasis. We noticed a significant increase in the proportion of stage III or IV patients in cluster A compared with cluster B. This suggests that there may be resistance to anoikis in cluster A, which could make cluster A patients more likely to develop lymph node or distant metastases. Cluster B, conversely, showed the opposite possibility. This distinction indicates that anoikis has distinct modes of action in CRC. We considered cluster A to be an anoikis-resistant cluster and cluster B an anoikis-activated cluster.

To explore this difference, we did a differential gene analysis of the two clusters. Intriguingly, some genes related to tumor development and progression showed a decreased expression in cluster B and increased expression in cluster A. In previous reports, FNDC1 and SFRP4 were closely linked to the development of epithelial-mesenchymal transition (EMT), which represents the ability to acquire migration^34^, ^35^. Further, knockdown of FNDC1 was found to inhibit proliferation, invasion and migration of gastric cancer cells, and by modulating the Wnt/β-catenin signaling pathway, FNDC1 may facilitate the EMT of gastric cancer cells^34^. Not coincidentally, SFRP4 expression was also found to be proportional to tumor invasion in gastric cancer, but the exact mechanism has not been elucidated^35^. In addition, Bin et al. found by bioinformatics methods that a high THBS2 expression was associated with a shortened survival, and this alteration was associated with the PI3K-AKT pathway and ECM. The authors thus considered it a potential biomarker of CRC^36^. Jie et al. further found that the knockdown of OLFM4 was able to promote metastasis of gastric cancer cells by activating the NF-κB/IL-8 axis^37^. Such an altered gene expression may promote the activation of anoikis in cluster B and prevent tumor metastasis.

We also noticed differences in functional enrichment. We are concerned that many ECM-related pathways are enriched. The normal cell survival is signaled by cell adhesion to ECM components as well as soluble growth factors and cytokines, while inappropriate ECM attachment or loss of cellular machinery anchoring is a key mechanism causing anoikis^38^. Failure to execute the anoikis program may lead to adnexal cells at the site of initial contact between matrix proteins and cell proliferation at ectopic sites where the stromal proteins are different from the stromal proteins^39^. This deregulation is an important hallmark of metastatic cancer cells. Our findings suggest that this resistance may emerge in CRC as a noteworthy research direction.

In cluster A, we discovered the increased expression of a number of pathways that have been reported in cancer metastasis studies. Jackstadt et al. found that the NOTCH signaling pathway promotes tumor metastasis by creating a microenvironment that attracts neutrophils^40^. The relationship between NOTCH signaling and anoikis has been reported in cervical tumors and prostate cancer. In cervical tumors, the activation of NOTCH signaling has been found to promote tumor metastasis by activating the PKB/Akt pathway and generating resistance to anoikis^41^. In prostate cancer, increased NOTCH signaling can inhibit anoikis in prostate epithelial cells by enhancing NF-κB activity^42^.

Moreover, the MAPK signaling pathway has been implicated in CRC metastasis^43^. This pathway is crucial for the regulation of cell-cell and cell-matrix contacts, and its disruption in the initiation of the anoikis program results in the death of non-normal cells, such as cancer cells^8^. The TGF-β pathway is an important pathway in the development of metastasis in CRC and has been reported to be linked to the loss of SMAD4, a transcription factor in TGF-β superfamily signaling that promotes tumor growth^44^. In previous reports, its association with anoikis resistance has also been suggested. Zou et al. reported that INHBB, a TGF family protein inhibits anoikis resistance and metastasis in nasopharyngeal carcinoma cells through attenuating the action of the TGF-β pathway^45^. In some CRC cases, TGF-β signaling, by increasing anoikis resistance, may enhance the ability of CRC cells to invade and colonize a second site^46^.

In the study, the expression of the TGF-β, MAPK and NOTCH signaling pathways was significantly increased in cluster A, further suggesting the presence of anoikis resistance in cluster A patients. These pathways were indeed reported to be associated with CRC metastasis in previous studies. Whether or not these pathways mediate anoikis resistance in CRC needs to be further investigated.

Changes in the TME are closely related to tumor metastasis^47^. In our immune analysis, anoikis resistance may be associated with immune activation. We also focused on the increased infiltration of MDSCs, macrophages and regulatory T cells in cluster A. In a previous report, macrophages in neoplasms were shown to drive angiogenesis and promote tumor cell migration and invasion 48. In hepatocellular carcinoma (HCC), M2 macrophages promote HCC cell migration and EMT via the TLR4/STAT3 signaling pathway^49^. In breast cancer, macrophage abundance positively correlates with EMT marker^50^. Such a relationship has also been reported in CRC. Tumor-associated macrophages (TAMs) can induce EMT by regulating the JAK2/STAT3/miR-506-3p/FoxQ1 axis to enhance CRC migration and invasion^51^. In addition, MDSCs not only suppress the immune system during tumor progression, but they also accelerate tumor growth, metastasis, and angiogenesis through VEGF^52^. Tregs show interaction with MDSCs in cancer, and the activation of Tregs by MDSCs is mainly caused by cytokines, such as IL-10 and TGF-β, which are also associated with the induction of MDSCs^53^. In CRC, the presence of a large number of Tregs reportedly predicts a poor prognosis 54. In our study, in the immune-activated microenvironment of cluster A, a large number of MDSCs, M2s and other immune cells were highly expressed, and such changes may be involved in the formation of anoikis resistance and promote tumor survival and metastasis.

CRC gene mutation research has been hot recently. The differences between the high- and low-risk groups may be related to the anoikis-related genes mutation. We focused on the BRAF gene mutation group, which had a higher risk score than the BRAF wild group. BRAF mutations can affect the MEKERK pathway, allowing cancer cells to survive in the absence of integrin-involved survival signals^39^, ^55^. This relationship has been previously demonstrated in melanoma. Patankar et al. found that BRAF mutations induce anoikis resistance in CRC, suggesting that this mechanism is likely generated by activation of the accessible database and needs independent prognostic cohort validation. Second, more tests are required to verify the involvement of anoikis in CRC,suggesting that this mechanism is likely generated by activation of the RAS-RAF-ERK pathway^56^.

Finally, the six selected genes (SIRT3, PIK3CA, ITGA3, DAPK1, PAK1, and CASP3) have been reported in colorectal cancer. PIK3CA is considered to be an oncogene in the PI3K pathway in colorectal cancer. Its involvement in colorectal cancer initiation is attributed to the upregulation of death-domain-associated protein, establishing a positive feedback regulatory relationship that promotes colorectal cancer progression^57^. Conversely, SIRT3 plays a crucial role in colorectal cancer, acting as the primary mitochondrial deacetylase. Its silence results in a reduction of mitochondrial biogenesis and dysfunction, exacerbating oxidative stress, reducing antioxidant defenses, and enhancing the effectiveness of oxaliplatin treatment^58^, ^59^. This suggests that SIRT3 may serve as a therapeutic target in colorectal cancer.DAPK1 is almost unexpressed in poorly differentiated colorectal cancer cells and gradually decreases at the invasive front of colorectal cancer, further emphasizing its critical role in diminishing tumor cell migratory capabilit,^60^, ^61^. In contrast, PAK1, encoding the serine/threonine p21-activated kinase, plays a role in KRAS-driven colorectal cancer cell proliferation. Its knockout leads to cell growth inhibition and apoptosis induction, highlighting its importance in colorectal cancer cell proliferation^62^. ITGA3, a member of the integrin alpha chain protein family, is associated with recurrence risk in right-sided colorectal cancer. Its involvement in colorectal cancer development is through influencing cell migration and reactive oxygen species generation^63^. Finally, CASP3 gene knockout manifests as a reduction in the epithelial-mesenchymal transition (EMT) phenotype in colorectal cancer cells, including an increase in E-cadherin expression and a decrease in the expression level of N-cadherin ZEB1^64^. This suggests that CASP3 loss may influence the biological characteristics of colorectal cancer by regulating the cell mesenchymal state, slowing down tumor cell invasion and metastasis. These genes play crucial roles in the regulatory network of colorectal cancer, providing important clues for a deeper understanding of the pathogenic mechanisms and therapeutic targets in colorectal cancer. And the prognostic significance of the model based on the six genes can be further strengthened after we further integrate the clinical features, and the predicted prognostic nomogram can effectively achieve individualized risk assessment.

Admittedly, our study has several limitations. First, our analysis is based on a publicly accessible database and needs independent prognostic cohort validation. Second, more tests are required to verify the involvement of anoikis in CRC.

## Conclusion

In summary, our study identified two distinct modes of anoikis in CRC, with active metastasis-promoting pathways inducing the formation of the anti-anoikis subtype, which has a stronger propensity for metastasis and a worse prognosis than an anoikis-activated subtype. We found that massive infiltration of immune cells may be an important marker for the formation of anoikis resistance. A systematic evaluation of anoikis patterns in CRC patients will facilitate our understanding of anoikis-related pathway mechanisms, cellular infiltration characteristics of the TME and the establishment of individualized therapy for CRC patients.

## Supplementary Material

Supplementary figures and tables.
