# Supplementary material for: Anoikis-related signature identifies tumor microenvironment landscape and predicts prognosis and drug sensitivity in colorectal cancer
Source: J Cancer. 2024 Jan 1;15(3):841–57. doi: 10.7150/jca.91627 (PMC10777033; doi:10.7150/jca.91627)
Supplement: Supplementary file 1 — Supplementary figures and tables. [file jcav15p0841s1.pdf]

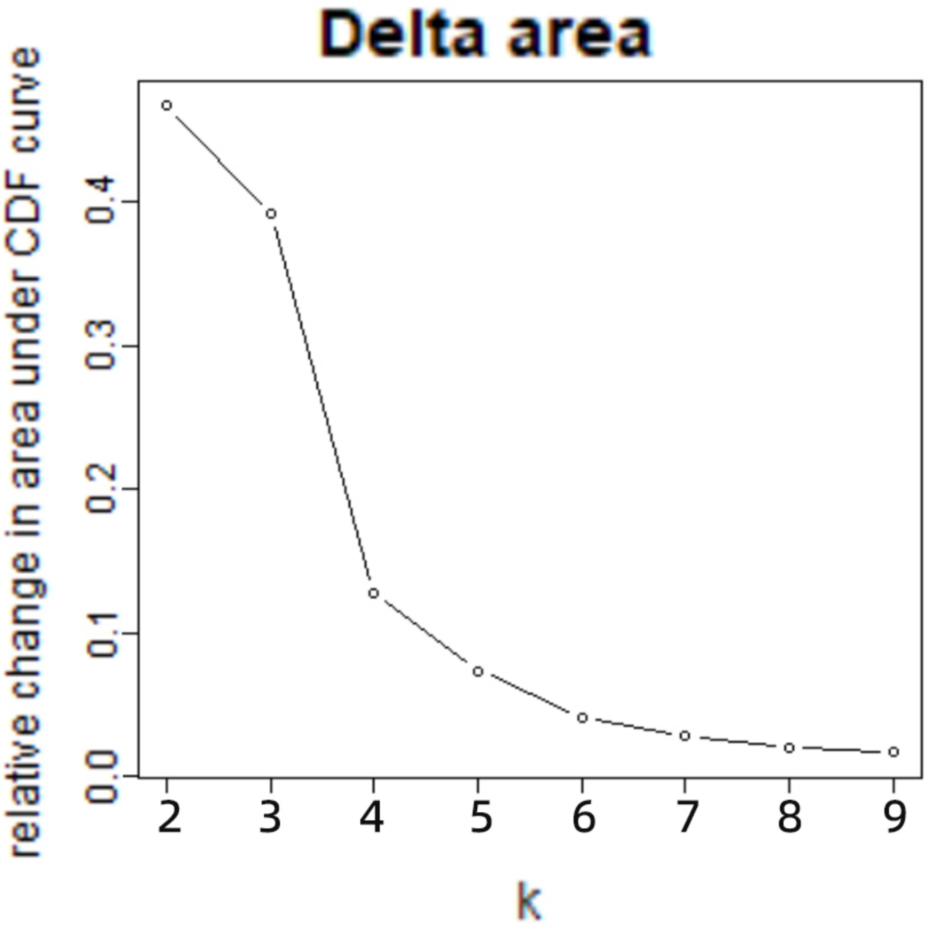
Delta area
relative change in area under CDF curve
0.0
0.1
0.2
0.3
0.4
2
3
4
5
6
7
8
9
k

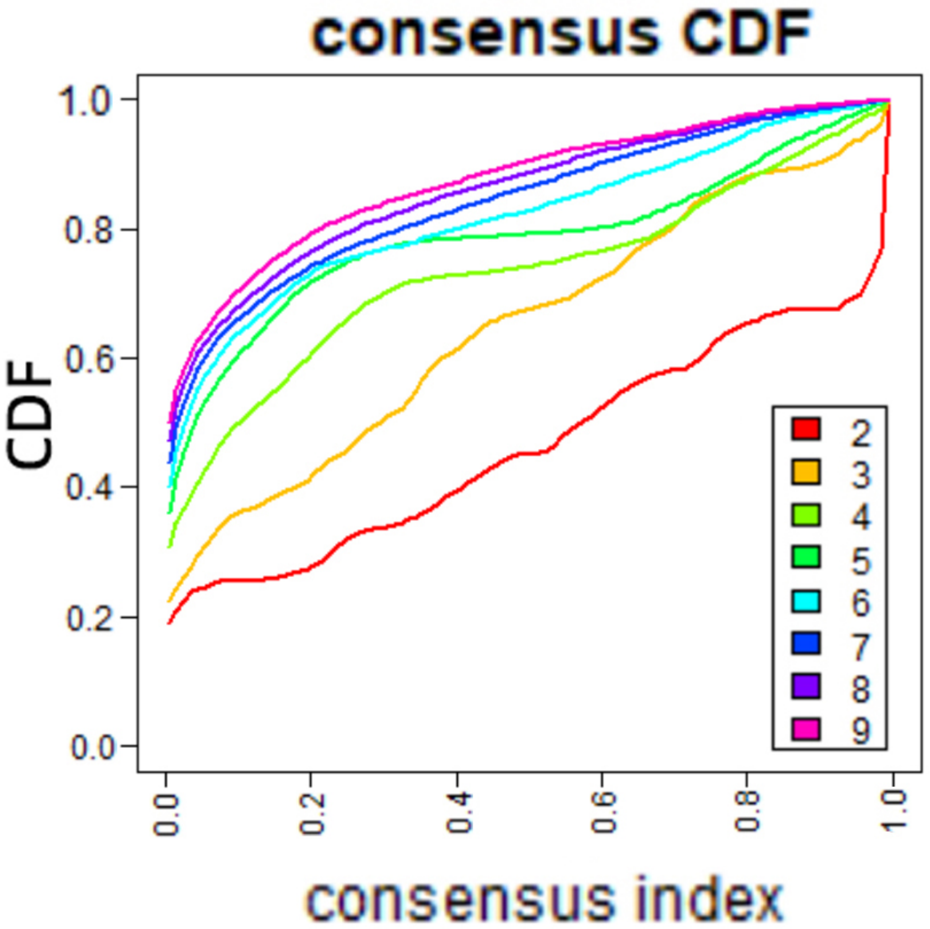
consensus CDF
CDF
0.0
0.2
0.4
0.6
0.8
1.0
2
3
4
5
6
7
8
9
consensus index

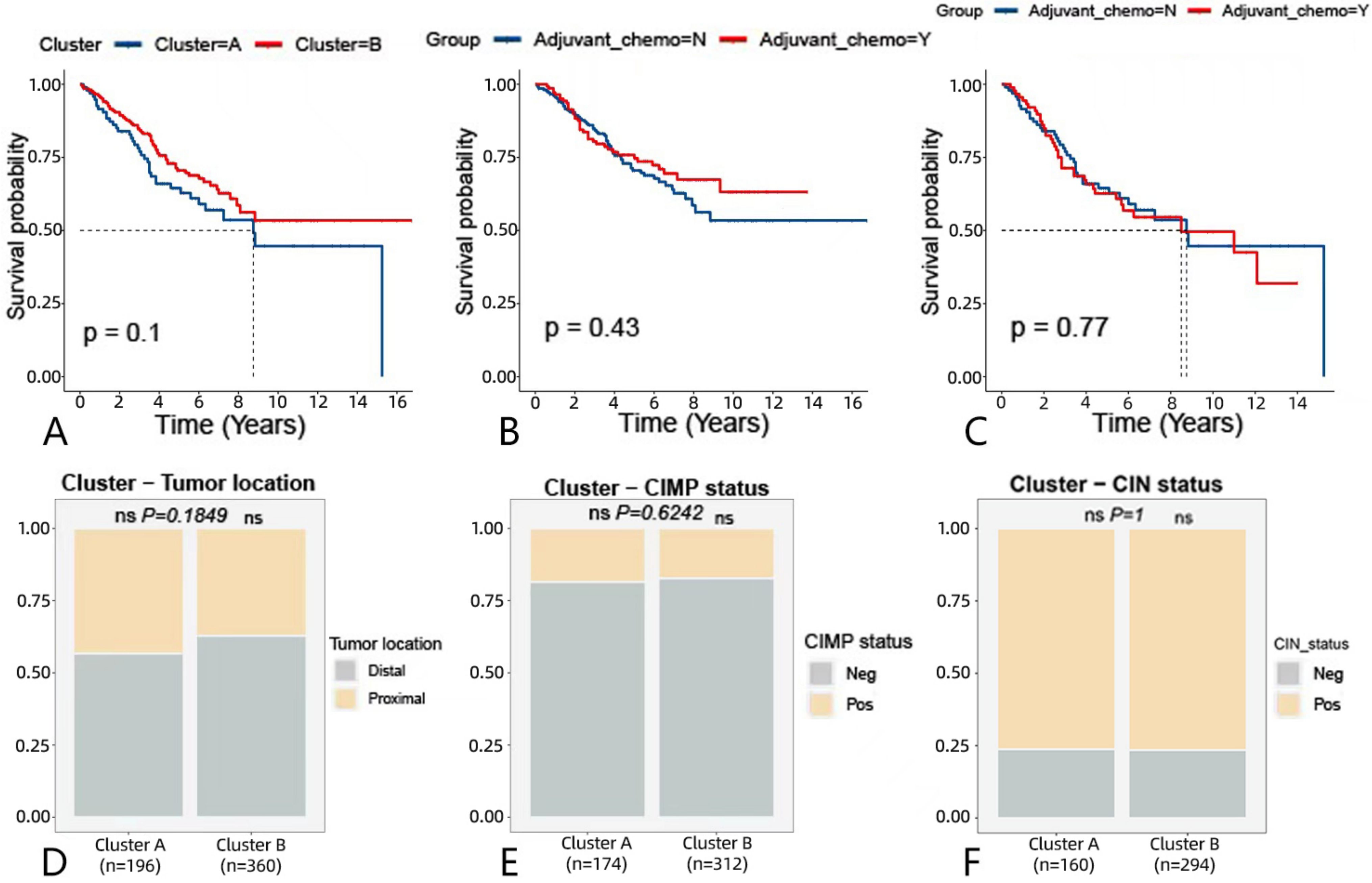
Cluster Cluster=A Cluster=B
Survival probability
1.00
0.75
0.50
0.25
0.00
p = 0.1
0 2 4 6 8 10 12 14 16
Time (Years)
A
Group Adjuvant_chemo=N Adjuvant_chemo=Y
Survival probability
p = 0.43
Time (Years)
B
Group Adjuvant_chemo=N Adjuvant_chemo=Y
Survival probability
p = 0.77
0 2 4 6 8 10 12 14
Time (Years)
C
Cluster – Tumor location
ns P=0.1849 ns
Tumor location
Distal
Proximal
Cluster A (n=196)
Cluster B (n=360)
D
Cluster – CIMP status
ns P=0.6242 ns
CIMP status
Neg
Pos
Cluster A (n=174)
Cluster B (n=312)
E
Cluster – CIN status
ns P=1 ns
CIN_status
Neg
Pos
Cluster A (n=160)
Cluster B (n=294)
F

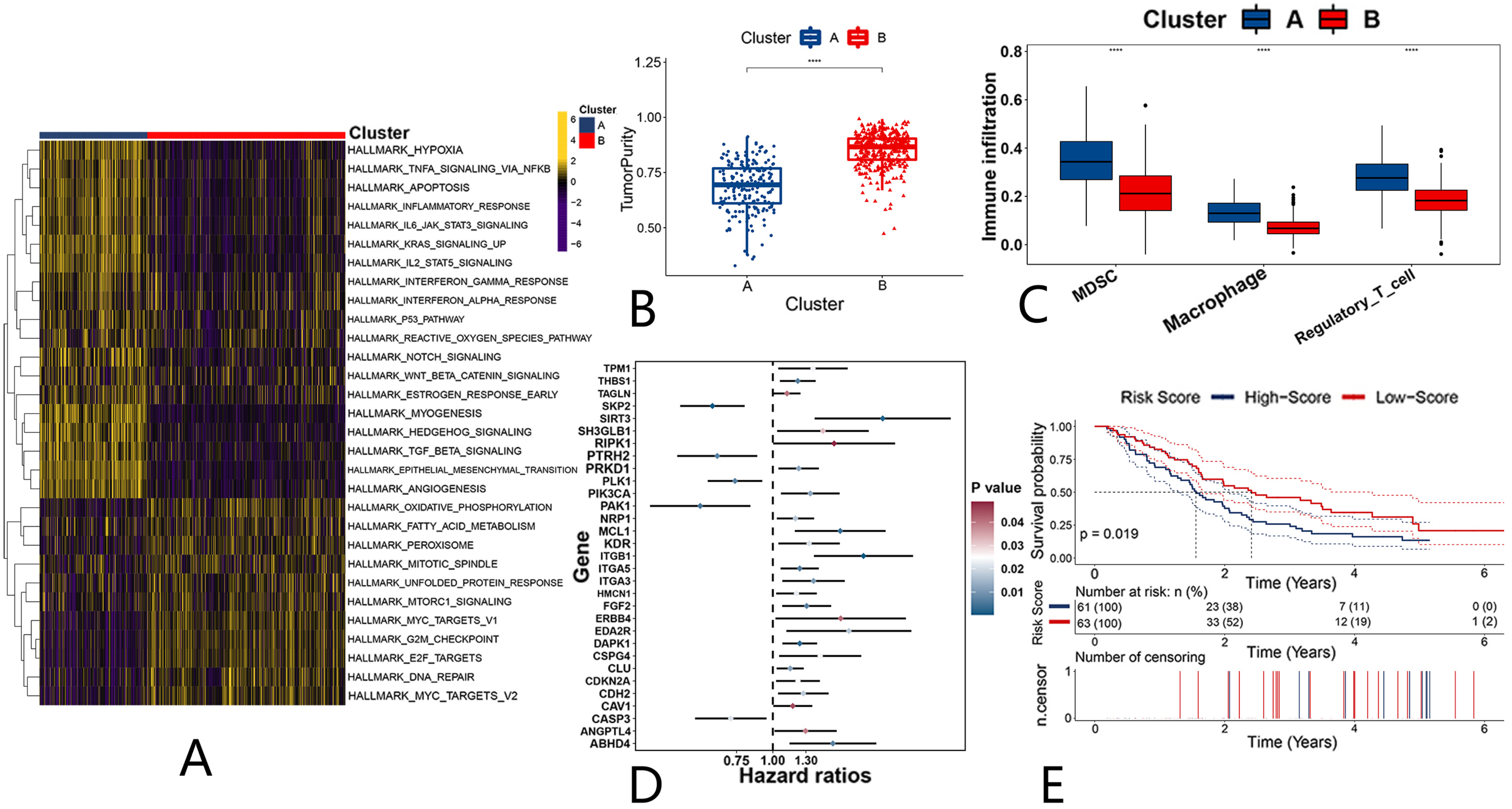

Cluster
HALLMARK_HYPOXIA
HALLMARK_TNFA_SIGNALING_VIA_NFKB
HALLMARK_APOPTOSIS
HALLMARK_INFLAMMATORY_RESPONSE
HALLMARK_IL6_JAK_STAT3_SIGNALING
HALLMARK_KRAS_SIGNALING_UP
HALLMARK_IL2_STAT5_SIGNALING
HALLMARK_INTERFERON_GAMMA_RESPONSE
HALLMARK_INTERFERON_ALPHA_RESPONSE
HALLMARK_P53_PATHWAY
HALLMARK_REACTIVE_OXYGEN_SPECIES_PATHWAY
HALLMARK_NOTCH_SIGNALING
HALLMARK_WNT_BETA_CATENIN_SIGNALING
HALLMARK_ESTROGEN_RESPONSE_EARLY
HALLMARK_MYOGENESIS
HALLMARK_HEDGEHOG_SIGNALING
HALLMARK_TGF_BETA_SIGNALING
HALLMARK_EPITHELIAL_MESENCHYMAL_TRANSITION
HALLMARK_ANGIOGENESIS
HALLMARK_OXIDATIVE_PHOSPHORYLATION
HALLMARK_FATTY_ACID_METABOLISM
HALLMARK_PEROXISOME
HALLMARK_MITOTIC_SPINDLE
HALLMARK_UNFOLDED_PROTEIN_RESPONSE
HALLMARK_MTORC1_SIGNALING
HALLMARK_MYC_TARGETS_V1
HALLMARK_G2M_CHECKPOINT
HALLMARK_E2F_TARGETS
HALLMARK_DNA_REPAIR
HALLMARK_MYC_TARGETS_V2
Cluster A B
6 4 2 0 −2 −4 −6
A
Cluster A B
TumorPurity
1.25 1.00 0.75 0.50
****
Cluster
B
Cluster A B
Immune infiltration
0.8 0.6 0.4 0.2 0.0
MDSC
Macrophage
Regulatory_T_cell
C
TPM1
THBS1
TAGLN
SKP2
SIRT3
SH3GLB1
RIPK1
PTRH2
PRKD1
PLK1
PIK3CA
PAK1
NRP1
MCL1
KDR
ITGB1
ITGA5
ITGA3
HMCN1
FGF2
ERBB4
EDA2R
DAPK1
CSPG4
CLU
CDKN2A
CDH2
CAV1
CASP3
ANGPTL4
ABHD4
Gene
P value
0.04 0.03 0.02 0.01
0.75 1.00 1.30
Hazard ratios
D
Risk Score High−Score Low−Score
Survival probability
1.00 0.75 0.50 0.25 0.00
p = 0.019
Time (Years)
Number at risk: n (%)
Risk Score
61 (100) 23 (38) 7 (11) 0 (0)
63 (100) 33 (52) 12 (19) 1 (2)
Number of censoring
n.censor
E

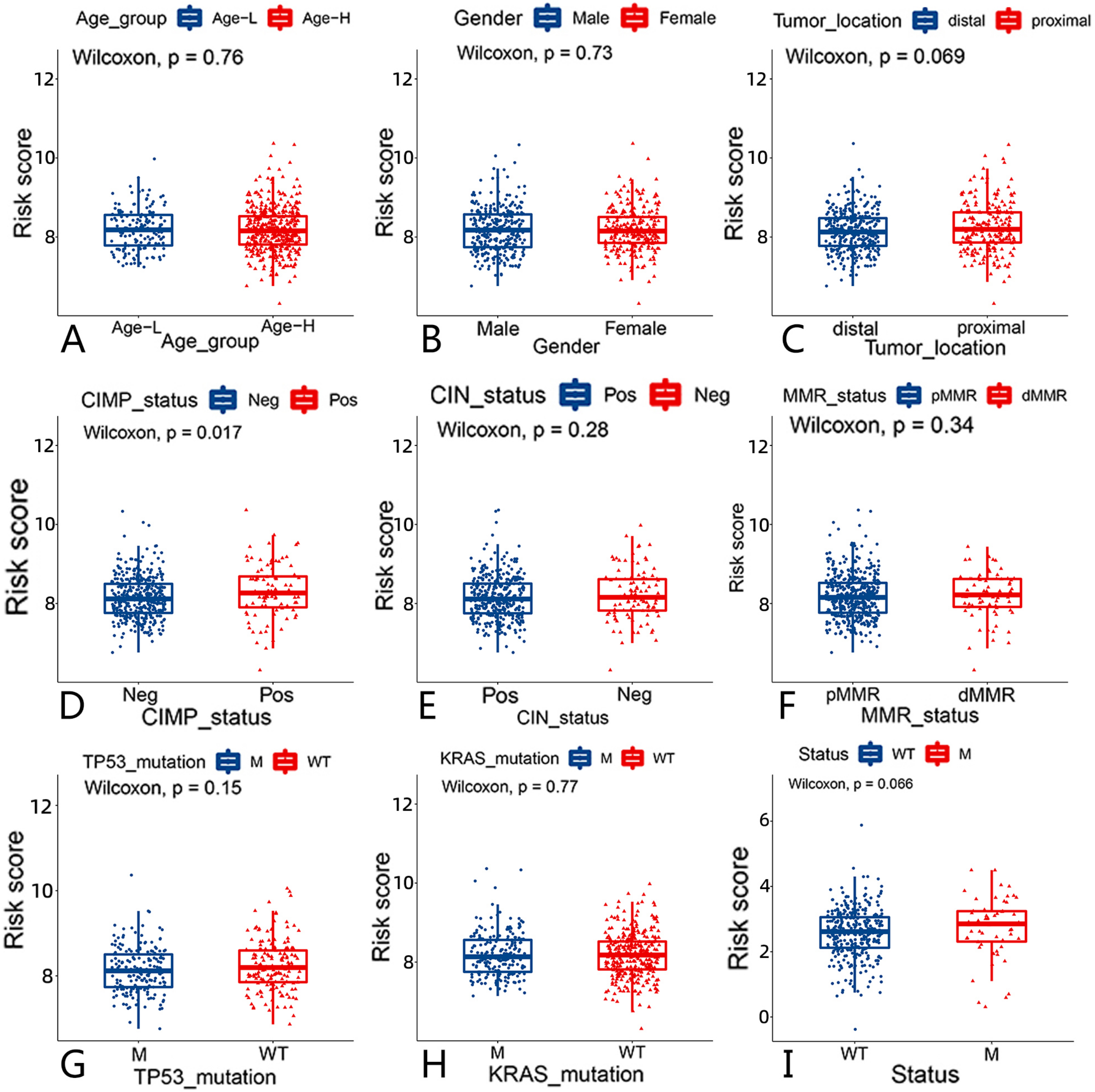
Age_group Age−L Age−H
Wilcoxon, p = 0.76
Risk score
Age_group
A
Gender Male Female
Wilcoxon, p = 0.73
Gender
B
Tumor_location distal proximal
Wilcoxon, p = 0.069
Tumor_location
C
CIMP_status Neg Pos
Wilcoxon, p = 0.017
CIMP_status
D
CIN_status Pos Neg
Wilcoxon, p = 0.28
CIN_status
E
MMR_status pMMR dMMR
Wilcoxon, p = 0.34
MMR_status
F
TP53_mutation M WT
Wilcoxon, p = 0.15
TP53_mutation
G
KRAS_mutation M WT
Wilcoxon, p = 0.77
KRAS_mutation
H
Status WT M
Wilcoxon, p = 0.066
Status
I

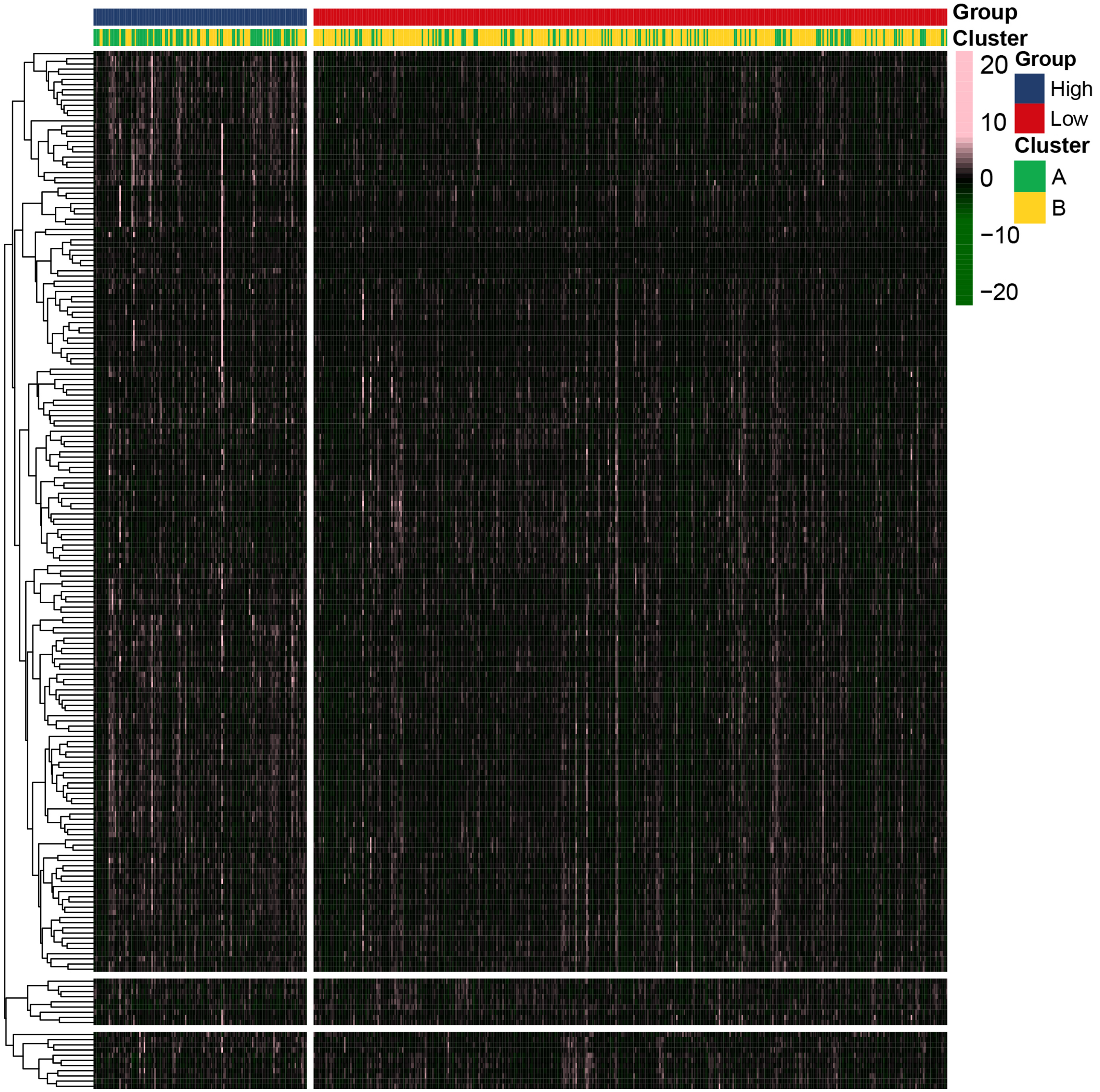
Group
Cluster
20
10
0
−10
−20
Group
High
Low
Cluster
A
B

	genes		genes		genes
1	YWHAZ	49	NRP1	97	CXCL12
2	XIAP	50	MYBBP1A	98	CTTN
3	WNT2	51	MTOR	99	CTNND1
4	WISP3	52	MTA1	100	CSPG4
5	USP9X	53	MMP2	101	CSNK2A1
6	UCHL1	54	MMP13	102	COPS5
7	TPM1	55	MMP11	103	CMA1
8	TP53	56	MGAT5	104	CLU
9	TIMP1	57	MET	105	CLDN1
10	THBS1	58	MDM2	106	CHUK
11	TGFB1	59	MCL1	107	CEBPB
12	TAGLN	60	MAVS	108	CEACAM6
13	STK11	61	MAPK3	109	CDKN2A
14	STAT3	62	MAPK1	110	CDH2
15	SRC	63	LTB4R2	111	CDH1
16	SNAI2	64	LRP1	112	CDCP1
17	SMAD4	65	LGALS1	113	CD63
18	SLCO1B3	66	KRAS	114	CCAR2
19	SKP2	67	KL	115	CAV1
20	SIRT3	68	KDR	116	CASP8
21	SIRPA	69	ITGB1	117	CASP3
22	SIK1	70	ITGAV	118	CASP2
23	SH3GLB1	71	ITGA8	119	CALR
24	SCRIB	72	ITGA6	120	BSG
25	S100A4	73	ITGA5	121	BRCA2
26	ROCK1	74	ITGA4	122	BRAF
27	RIPK1	75	ITGA3	123	BMF
28	RHOC	76	ITGA2	124	BIRC3
29	RHOA	77	IKZF3	125	BCL2L11
30	RAD9A	78	IGF1R	126	BCL2
31	PTRH2	79	HTRA1	127	BCAR1
32	PTPN11	80	HOXA10	128	BAX
33	PTK6	81	HMGA1	129	ANGPTL4
34	PTK2B	82	HMCN1	130	AKT2
35	PTK2	83	HK2	131	AKT1
36	PTHLH	84	HGF	132	AFAP1L1
37	PTEN	85	FN1	133	ABHD4
38	PRKD1	86	FGF2		
39	PRKCA	87	FER		
40	PLK1	88	ERBB4		
41	PIK3CG	89	EIF2AK3		
42	PIK3CA	90	EGFR		
43	PECAM1	91	EEF2K		
44	PAK4	92	EEF1A1		
45	PAK1	93	EDA2R		
46	OLFM3	94	DLG1		
47	NTRK2	95	DAPK1		
48	NTF3	96	DAP3		

Gene	Species	Primer sequence (5′ to 3′)
PIK3CA	human	sense primer:CCAATCCCAGGTGGAATGAA
		antisense primer:CCTTTCGGCCTTTAACAGAGC
SIRT3	human	sense primer:TGCTTCTGCGGCTCTACACG
		antisense primer:ACGTCAGCCCGAATGTCCTC
DAPK1	human	sense primer:GATTACTACGACACCGGCGA
		antisense primer:ATTTGGCGGCATACTGGAGG
PAK1	human	sense primer:AGAGCCTTGTACCTCATTGCC
		antisense primer:GGAATTGATGCAGCTATCATGGA
ITGA3	human	sense primer:TTCAAACGGAACCAGAGGATG
		antisense primer:GCTAAGCGAGGTCTGGAGTGTAT
CASP3	human	sense primer:AGAACTGGACTGTGGCATTGAG
		antisense primer:CACAAAGCGACTGGATGAACC
